# Out-of-hours discharge from critical care: does it matter?

**DOI:** 10.1186/cc14606

**Published:** 2015-03-16

**Authors:** M Ahmed, G Lumley, S Nourse, A Hurding, A Thomas, M Healy

**Affiliations:** 1 The Royal London Hospital, London, UK

## Introduction

The aim of this audit was to assess the clinical impact of out-of-hours (OOH) discharge from the adult critical care unit (ACCU) in a tertiary care hospital. Discharging patients from critical care OOH has been associated with both higher mortality and increased rate of readmissions [1,2]. As a result, national guidance stipulates that OOH discharges from critical care should be avoided [3].

## Methods

A retrospective snapshot analysis of patients was conducted at least 48 hours after discharge from the ACCU in October 2014. Patients discharged OOH (20:00 to 07:59) were compared with those discharged in hours (IH; 08:00 to 19:59). Analysis included: patient demographics, APACHE II score, handover procedure prior to discharge, follow-up by the receiving team, appropriate and timely drug prescribing, recognising and acting upon clinical deterioration and readmission rates.

## Results

A total of 161 patients were discharged from the ACCU in October 2014. Of these, 46% of the patients were discharged OOH. Forty-one (of 74) OOH and 19 (of 87) IH discharges were sampled for further analysis. The baseline demographics and APACHE II score were similar between both groups. The majority of discharges were delayed (>4 hours, 90% IH vs. 93% OOH). More patients had nursing handover completed if discharged IH (88% IH vs. 61% OOH) and doctors' summaries were less frequently completed OOH (83% OOH vs. 94.4% IH). A management plan for the ward was outlined in 94% of IH versus 65.% of OOH discharges. Seventy-eight per cent OOH versus 95% IH patients discharged were reviewed by a doctor within 24 hours. Twenty-nine per cent OOH versus 67% IH patients discharged were reviewed by a consultant within 24 hours. Following discharge a management plan was followed in 94% of IH patients versus 44% OOH, patients had drug charts correctly charted in 100% of IH cases versus 66% OOH and missed/delayed doses were documented in 11.1% of IH cases versus 61% OOH. There was no difference between the groups in incidence of clinical deterioration and recognition and follow-up of clinical deterioration. Two patients were readmitted within 48 hours from the OOH group.

## Conclusion

This audit compares with current evidence suggesting harm from OOH discharge despite most discharges being delayed. Discharging patients is a complex hospital process, but focus needs to be on discharging patients IH. Therefore, areas for improvement include targeting forward flow of patients throughout the hospital, completion of handover documents IH and publication of guidance for receiving teams.
